# The Hierarchical Contribution of Organic vs. Conventional Farming, Cultivar, and Terroir on Untargeted Metabolomics Phytochemical Profile and Functional Traits of Tomato Fruits

**DOI:** 10.3389/fpls.2022.856513

**Published:** 2022-03-25

**Authors:** Gabriele Rocchetti, Biancamaria Senizza, Gokhan Zengin, Paolo Bonini, Luana Bontempo, Federica Camin, Marco Trevisan, Luigi Lucini

**Affiliations:** ^1^Department of Animal Science, Food and Nutrition, Università Cattolica del Sacro Cuore, Piacenza, Italy; ^2^Department for Sustainable Food Process, Università Cattolica del Sacro Cuore, Piacenza, Italy; ^3^Department of Biology, Faculty of Science, Selcuk University, Konya, Turkey; ^4^OloBion–OMICS LIFE LAB, Barcelona, Spain; ^5^Traceability Unit, Fondazione Edmund Mach, San Michele all’Adige, Italy; ^6^Center Agriculture Food Environment, University of Trento, San Michele all’Adige, Italy

**Keywords:** *Solanum lycopersicum* L., organic farming, antioxidants, metabolomics, polyphenols, functional quality

## Abstract

In this work, the impact of terroir, cultivar, seasonality, and farming systems on functional traits of tomato was hierarchically investigated. Untargeted metabolomics, antioxidant capacity, colorimetric assays, and enzyme inhibition were determined. The total phenolic and carotenoid contents significantly varied between growing years, whereas an interaction between the farming system and growing year (*p* < 0.01) was observed for total phenolics, carotenoids, and flavonoids, and for acetylcholinesterase inhibition. Hierarchical clustering showed that geographical origin and growing year were the major contributors to the differences in phytochemical profiles. Nonetheless, supervised modeling allowed highlighting the effect of the farming system. Several antioxidants (L-ascorbic acid, α-tocopherol, and 7,3′,4′-trihydroxyflavone) decreased, whereas the alkaloid emetine and phytoalexin phenolics increased under organic farming. Taken together, our findings indicate that cultivar and pedo-climatic conditions are the main determinants for the functional quality of tomato, whereas the farming system plays a detectable but hierarchically lower.

## Introduction

Tomato (*Solanum lycopersicum* L.) is one of the most cultivated and consumed crops worldwide, and a major source of bioactive compounds to the human diet, with an essential role in the prevention of cardiovascular diseases and cancer (Szabo et al., 2018). These beneficial properties are mainly associated with a broad spectrum of compounds, such as organic acids, polyphenols, terpenes, proteins, vitamins, and carotenoids. Among these latter, lycopene represents 80–90% of the total content, followed by beta-carotene, phytoene, phytofluene, neurosporene, and lutein. Regarding the class of phenolic compounds, tomato is mainly characterized by the flavonoids rutin, naringenin, naringenin chalcone and quercetin, and the hydroxycinnamic acids chlorogenic and caffeic acids (Rocchetti et al., 2019).

In the last years, the consumer’s awareness about food quality and food safety led to increasing demand for fresh organic products because they are considered of higher nutritional value and improved contents of functional compounds, with significantly lower chemicals residue. In this regard, health-related issues are important factors influencing the consumers’ market preferences. Previous studies based on the comparisons between organic and conventional farming systems highlighted a higher nutritional value in crops from organic farming (Hallmann, 2012), but the results are still contradictory, and no proper evidence to recommend organic over conventionally are stated. Johansson et al. (2014) showed that the carotenoid and phenolic contents of different crops (including tomato) could be affected by different factors, such as crop, year, environment, and harvest time, in addition to the specific farming system considered. Similarly, the availability and the type of fertilizers can modulate the biosynthetic pathways of plants and consequently the food composition (Novotná et al., 2012). In general, the wider concept of terroir accounts for the modulation of phytochemical profiles in plant-based foods (Lucini et al., 2020). Notwithstanding, Hallmann (2012) previously reported a higher content of polyphenols and ascorbic acid under organic farming. However, only some specific compounds (such as chlorogenic acid, quercetin, and rutin) have been enhanced under organic farming (Hallmann et al., 2013). These findings were in accordance with a previous study by Mitchell et al. (2007), who demonstrated that organic tomato possessed a higher content of quercetin and naringenin than the conventional. Additionally, Riahi et al. (2009) found a non-significant correlation between the carotenoid profile of tomato and its growing system. Other authors reported contradictory results; for example, Rossi et al. (2008) showed a decrease in lycopene content in tomato according to organic farming conditions, while Caris-Veyrat et al. (2004) recorded on the same crop a significantly higher total carotenoid content.

Contradictory and still fragmented information can be found on vegetables from organic agriculture, probably because of the crosstalk between farming systems and pedo-climatic conditions, seasonal variability, as well as specific responses related to the different biochemical pathways and cultivar-related responses. Therefore, in this work, an untargeted metabolomic approach for phenolic profiles and a set of other functional traits were used to compare tomatoes cultivated under either organic or conventional conditions. The experimental design included several other factors (cultivars, terroir, seasonality) potentially affecting tomato functional properties, to comparatively investigate their actual impact. The final aim was to hierarchically investigate the contribution of different factors on the functional quality of organic or conventional tomato.

## Materials and Methods

### Plant Materials and Cultivation

In this work, the impact of terroir and farming systems was considered by accounting: two growing years (2012 and 2013); two tomato cultivars (San Marzano, Round, and Roma, Long); two farming systems (organic and conventional); two different regions (Emilia-Romagna and Basilicata, Northern and Southern Italy respectively). Samples were obtained directly from farms through on-farm trials; each sample was made by pooling 1 kg of berries randomly harvested from a minimum of 15 plants in a 50 m^2^ plot, for each experimental condition (per specific origin, cultivar, year, and farming system). Plants grown within the organic regime were managed in full compliance with the EU regulations for organic farming (European Community Council Regulation EC 834/2007), and the fields involved have been managed organically for more than 10 years. The field sites were managed according to the common agricultural practice for tomato in Italy, were all based on stockless crop production, but local agronomic practices differed between Emilia-Romagna and Basilicata depending on to climate and soil types. All the details of the farming conditions, as well as average climatic conditions in both field sites, are provided as Supplementary Material. In both the farming systems, the plants were irrigated. The conventional fields were provided with both organic and mineral fertilizers and with chemical weed protection agents. The organic fields were supplied only with organic fertilizers and the weed management was treated mechanically. All details of the management of the plants are reported in Bontempo et al. (2020), while a comprehensive list of the samples origin, cultivar type, farming system, and growing year can be found in Table 1.

**TABLE 1:** List of the different tomato samples under investigation.

ID	Origin	Cultivar	Farming system	Growing year
Sample 1	Basilicata	Round	Organic	2012
Sample 2	Basilicata	Round	Conventional	2012
Sample 3	Basilicata	Long	Organic	2012
Sample 4	Basilicata	Long	Conventional	2012
Sample 5	Emilia Romagna	Round	Organic	2012
Sample 6	Emilia Romagna	Round	Conventional	2012
Sample 7	Emilia Romagna	Long	Organic	2012
Sample 8	Emilia Romagna	Long	Conventional	2012
Sample 9	Basilicata	Round	Organic	2013
Sample 10	Basilicata	Round	Conventional	2013
Sample 11	Basilicata	Long	Organic	2013
Sample 12	Basilicata	Long	Conventional	2013
Sample 13	Emilia Romagna	Round	Organic	2013
Sample 14	Emilia Romagna	Round	Conventional	2013
Sample 15	Emilia Romagna	Long	Organic	2013
Sample 16	Emilia Romagna	Long	Conventional	2013

### Extraction and Ultra-High-Performance Liquid Chromatography-Quadrupole-Time-of-Flight Mass Spectrometry Analysis

The lyophilized samples were extracted using a material to solvent ratio of 1:10 (w/v). The extraction solvent consisted of a mixture of 80% aqueous methanol acidified with 0.1% formic acid. Samples were mixed by vortexing for 3 min, ultrasonic processed for 5 min, and then a homogenizer-assisted extraction based on Ultra-turrax (Ika T25, Staufen, Germany) at 5,000 × *g* for 3 min was done. The extracts were then centrifuged (Eppendorf 5810R, Hamburg, Germany) at 7,000 × *g* for 10 min at 4°C. Finally, samples were filtered using 0.2 μm cellulose syringe filters and the resulting solutions were collected in amber vials until further analysis.

In this work, the identification of the different tomato metabolites was made using a metabolomics-based approach. We used ultra-high-performance liquid chromatography (UHPLC) coupled with a quadrupole-time-of-flight (QTOF) mass spectrometry, as previously reported (Rocchetti et al., 2020). Briefly, the separation was achieved through an Agilent Zorbax Eclipse Plus C18 column (50 × 2.1 mm, 1.8 μm), with water-acetonitrile linear gradient elution from 6 to 94% acetonitrile in 32 min. Samples were analysed in positive polarity (ESI +), using a full scan mode in a typical *m/z* range of 100–1,200, with a scan rate of 0.8 spectra/s and a nominal mass resolution of 30,000 FWHM. Also, pooled quality control samples were randomly injected throughout the sequence and analysed in data-dependent auto-MS/MS mode, using 10 precursors per scan cycle and typical collision energies of 10, 20, and 40 eV. Also, to avoid possible experimental bias, the injections sequence was randomized, and blank samples (extraction solvent only) were included every ten injections.

### Data Processing

The raw MS data were processed using the software Agilent Profinder B.06 software (from Agilent Technologies), according to the targeted find-by-formula algorithm, exploiting the information of monoisotopic mass and isotope profile of each mass feature for annotation purposes. The annotation process followed mass and retention time alignment, adopted a 5-ppm tolerance for mass accuracy, and corresponded to level 2, putative identification, according to Cosmos coordination of standards in metabolomics. The database built combining Phenol-Explorer^1^ and Food-DB (considering the chemical composition reported for tomato)^2^ was used. Also, structural confirmation of metabolites was carried out from tandem MS data of the pooled quality control samples using the MS-Dial workflow, considering the comprehensive experimental spectra database Mass Bank of North America (MoNA) (Tsugawa et al., 2015).

### Analysis of Total Phenolics, Total Flavonoids, and Total Carotenoids

Spectrophotometric methods were used to determine total phenolic and flavonoid content, as conducted previously Uysal et al. (2016). Gallic acid (GAE) for phenolic and rutin equivalent (RE) were used for phenolics and flavonoid, respectively. Total carotenoids were determined colorimetrically, without saponification, based on the mean absorption coefficients and mean absorption wavelength.

### *In vitro* Antioxidant and Enzyme Inhibition Properties

The antioxidant protocols included reducing power, namely cupric reducing antioxidant capacity (CUPRAC) and ferric reducing power (FRAP), metal chelating (MCA), phosphomolybdenum (PBD), and free radical scavenging [2,2-diphenyl-1-picrylhydrazyl (DPPH) and 3-ethylbenzothiazoline-6-sulphonic acid (ABTS)] activities. Experimental details were as described previously by Uysal et al. (2016). Trolox and ethylenediaminetetraacetic acid (EDTA) were used to express radical scavenging and metal chelating antioxidant assay, respectively. Finally, the inhibitory effects of the extracts were tested against different enzymes, such as tyrosinase, α-amylase, acetylcholinesterase (AChE), and butyrylcholinesterase (BChE). Galantamine for cholinesterases, kojic acid for tyrosinase, and acarbose for α-amylase were used to express enzyme inhibitory results. Experimental details were as described previously by Uysal et al. (2016).

### Statistical Analysis

The effects of terroir, growing year, cultivar, farming systems, and their interactions on the measured parameters were evaluated with one-way and two-way ANOVA followed by Duncan’s *post-hoc* test (*p* < 0.05), using the software SPSS 26.0.

The metabolomic dataset resulting from the UHPLC-QTOF mass spectrometry analysis was used to provide univariate and multivariate statistical outputs. The statistical workflow was done using the online software MetaboAnalyst.^3^ The Volcano Plot analysis was done considering the possible pairwise comparison, as a function of the “cultivar type,” “growing year,” “geographical origin,” and “farming system,” using a Fold-Change (FC) cut-off > 1.2. After that, both unsupervised (i.e., hierarchical cluster analysis, HCA, and principal component analysis, PCA) and supervised (i.e., orthogonal projections to latent structures discriminant analysis, OPLS-DA) were carried out on the same software. The OPLS-DA prediction model was cross-validated using a CV-ANOVA (*p* < 0.01), excluding model overfitting by using a permutation plot (number of random permutations: 100). Also, the model’s parameters related to the goodness-of-fit (cumulative R^2^Y) and goodness-of-prediction (cumulative Q^2^) were recorded and checked. The variables importance in orthogonal projections (VIP) was then inspected by using a VIP score (i.e., discrimination power) cut-off > 1. Finally, the S-plot following the OPLS-DA model was used to provide those box plots of the most discriminant metabolites of each condition.

## Results and Discussion

### Phytochemical Profile of the Different Tomato Cultivars

In this work, untargeted metabolomics based on UHPLC-QTOF mass spectrometry allowed us to identify a total of 611 compounds (comprising 134 compounds confirmed by the MS/MS annotation workflow). In this regard, the approach used provided a putative identification of 380 phenolic compounds (included in Phenol-Explorer), followed by 97 compounds typical of tomato (as provided in the database FoodDB). As can be observed from the pie chart presented in Supplementary Figure 1, flavonoids, amino acids and peptides, phenolic acids (including benzoic acids, cinnamic acids, and phenylacetic acids), and benzamides were the most represented classes of compounds. A detailed list of the phytochemicals identified in the different sample replicates can be found in the Supplementary Table 1, together with their composite mass spectra and individual abundance values. Going into detail of the metabolomics dataset, hydroxycinnamic acids were largely represented (64 compounds), followed by flavones and flavanones (accounting for 53 compounds), flavonols (44 compounds), and lignans (26 compounds). FoodDB allowed annotating amino acids, phytosterols (such as beta-sitosterol and campesterol), tocopherols and isomeric forms of beta-carotene, L-ascorbic acid, and acetoxytomatine (Supplementary Table 1). The phytochemicals outlined by untargeted metabolomics were in accordance with the typical composition reported for tomato fruits that are known to be a good source of phenolics, carotenoids, vitamins, and glycoalkaloids (Chaudhary et al., 2018). The interest toward the phytochemical profile of different tomato cultivars lies in the potential health benefits associated with these bioactive constituents, including antioxidant, anti-mutagenic, anti-proliferative, anti-inflammatory, and anti-atherogenic activities (Chaudhary et al., 2018).

### Total Phenolics, Total Carotenoids, and *in vitro* Bioactive Properties

Table 2 reports the effect of the farming system, cultivar, origin, and growing year on the flavonoids and carotenoids content, *in vitro* antioxidant properties, and enzyme inhibitory capacities of the tomato samples under investigation. Noteworthy, the total amount of phenolic compounds and flavonoids in the tested samples was primarily influenced by the growing season. The level of these components in 2012 [Total phenolic contents (TPC): 16.24 mg GAE/g and total flavonoid content (TFC): 0.99 mg GAE/g] were higher than that of 2013 (TPC: 13.87 mg GAE/g and TFC: 0.69 mg RE/g) (*p* < 0.001 and *p* < 0.05). This observation can be related to weather conditions, especially temperatures, rainfall, and bright hours of sunshine. Consistently with our approach, previous works have reported that the levels of polyphenols significantly fluctuated over the growing seasons (Mabizela et al., 2020). As reported in Bontempo et al. (2020), 2012 was generally hotter than 2013 for both sites, a condition known to elicit the accumulation of phenolics together with other abiotic stress conditions (Jan et al., 2021). Noteworthy, we did not observe any significant difference in the tested tomato samples regarding TPC and TFC as a function of farming system, cultivar, and origin. Regarding total carotenoid content (TCC), we observed only a significant impact of the growing season (2012 *vs.* 2013), with higher values in 2013 (4.20 mg/100g DW) than in 2012 (1.27 mg/100 g DW). Interestingly, a significant (*p* < 0.001) effect of the interaction between the farming system and growing year was also observed for TCC (Table 2).

**TABLE 2:** Effect of the farming system (F), cultivar (C), origin (O), and growing year (G) on the phenolic and carotenoid contents, antioxidant activities and inhibitory activities of the different tomato samples, expressed as mg/g of dry weight.

		TPC	TCC	TFC	DPPH	ABTS	CUPRAC	FRAP	Phosp.	Metal Chelat.	AChE	BChE	Tyrosinase	α-amylase
Farming system (F)	*p*-value	0.726	0.610	0.434	0.892	0.555	0.866	0.815	0.748	0.412	0.973	0.600	0.196	0.735
	Conventional	15.30	2.58	0.74	12.70	24.77	32.45	19.70	1.01	16.04	2.11	2.87	53.41	0.22
	Organic	14.83	2.89	0.95	12.84	26.39	32.79	19.46	0.99	15.34	2.11	2.80	54.75	0.22
Cultivar (C)	*p*-value	0.118	0.784	0.127	0.219	0.187	0.273	0.074	0.013	0.939	0.912	0.159	0.353	0.537
	Round	14.49	2.80	0.95	12.41	24.59	32.01	19.06	0.96	15.67	2.11	2.76	54.39	0.21
	Long	15.63	2.67	0.73	13.13	26.57	33.23	20.09	1.04	15.70	2.10	2.90	53.75	0.22
Origin (O)	*p*-value	0.885	0.127	0.513	0.047	0.480	0.175	0.614	0.783	<0.001	<0.001	0.052	0.028	0.050
	Basilicata	15.00	2.37	0.89	12.19	25.05	31.86	19.73	0.99	14.54	2.19	2.93	53.33	0.21
	Emilia-Romagna	15.11	3.10	0.79	13.34	26.11	33.37	19.43	1.00	16.84	2.73	2.73	54.81	0.23
Growing year (G)	*p*-value	<0.001	<0.001	0.045	0.106	0.101	<0.001	0.367	0.246	<0.001	0.900	0.259	<0.001	0.837
	2012	16.24	1.27	0.99	13.24	26.80	34.39	19.84	0.98	14.77	2.10	2.77	52.93	0.22
	2013	13.87	4.20	0.69	12.29	24.35	30.84	19.31	1.02	16.60	2.11	2.89	55.22	0.22
*FxC*	*p*-value	0.829	0.871	0.411	0.174	0.408	0.805	0.570	0.937	0.129	0.404	0.961	0.410	0.038
*FxO*	*p*-value	0.134	0.368	0.030	0.150	0.249	0.116	0.117	0.277	0.500	0.903	0.290	0.738	<0.001
*FxG*	*p*-value	0.007	<0.001	<0.001	0.670	0.825	0.180	0.248	0.636	0.909	<0.001	0.059	0.235	0.278

*TPC, total phenolic content; TCC, total carotenoid content; TFC, total flavonoid content; Phosp., phosphomolybdenum activity; Metal Chelat., metal chelating activity.*

The *in vitro* antioxidant properties were then evaluated through different complementary approaches, namely radical quenching (ABTS and DPPH), reducing power (CUPRAC, FRAP, and phosphomolybdenum), and metal chelating assays. The antioxidant properties, particularly DPPH, CUPRAC, and metal chelating properties, were influenced by the sample’s origin and season of growth. For example, the reducing power in CUPRAC assays in the season 2012 (34.39 mg TE/g) was significantly higher than in 2013 (30.84 mg TE/g). Conversely, the metal chelating ability in the growth year 2013 (16.60 mg EDTAE/g) was stronger than in 2012 (14.77 mg EDTAE/g). In contrast to the TPC, most of the antioxidant capacities did not change between the two growing years. This result may be related to the possible contribution by non-phenolic antioxidants such as carotenoids, tocopherol, and vitamin C (Sharma et al., 2021) as well as synergistic or antagonistic interactions between them. Indeed, previous studies reported no clear correlations between total phenolics and *in vitro* antioxidant properties (Yu et al., 2021).

The cholinesterases (AChE and BChE), tyrosinase, and amylase enzyme inhibition assays we carried out are closely related to treating global health problems, such as diabetes mellitus, obesity, and Alzheimer’s disease. As provided in Table 2, the enzyme inhibitory capacities were significantly influenced by the origin. Except for BChE, samples from the Emilia-Romagna region showed higher inhibitory effects than the samples from Basilicata. For example, the samples from Emilia-Romagna showed a higher tyrosinase inhibitory effect with a value of 54.81 mg KAE/g compared to the samples from Basilicata (53.33 mg KAE/g). Like for antioxidant assays, the enzyme inhibitory abilities did not correlate directly with the total amounts of phenolics. This is in line with previous reports showing no correlation between phenolics and enzyme inhibitory abilities (Schmeda-Hirschmann et al., 2021). For example, alkaloids show more effects on cholinesterase than phenolic compounds (Ahmed et al., 2021). Overall, in both the antioxidant and enzyme inhibitory assays, the farming systems, and cultivars did not affect the observed results. On the contrary, the origin and growing year influenced the functional traits we observed. This fact shows that pedo-climatic conditions can have a major impact on our biological activities, compared to farming system and cultivar.

### The Combined Effect of Farming System, Cultivar, and Terroir on Phytochemical Profile

**FIGURE 1:**
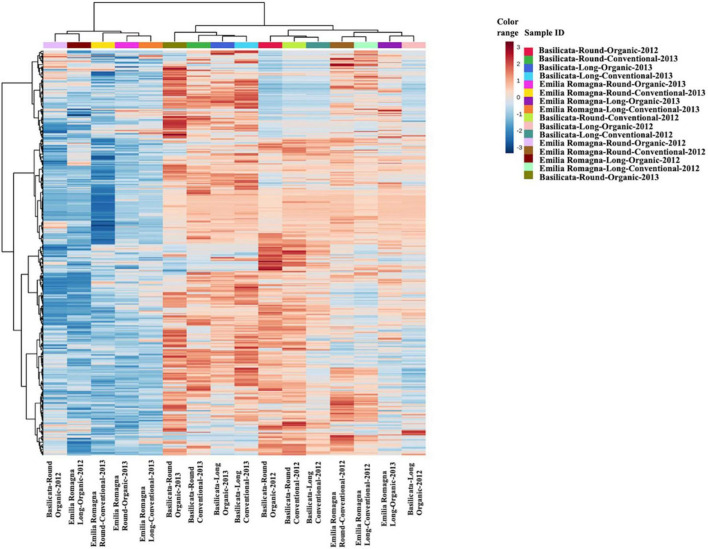
Unsupervised hierarchical cluster analysis (HCA) (Euclidean distance) relative to untargeted phytochemical profile of tomato samples according to terroir, cultivar, and farming systems.

**FIGURE 2:**
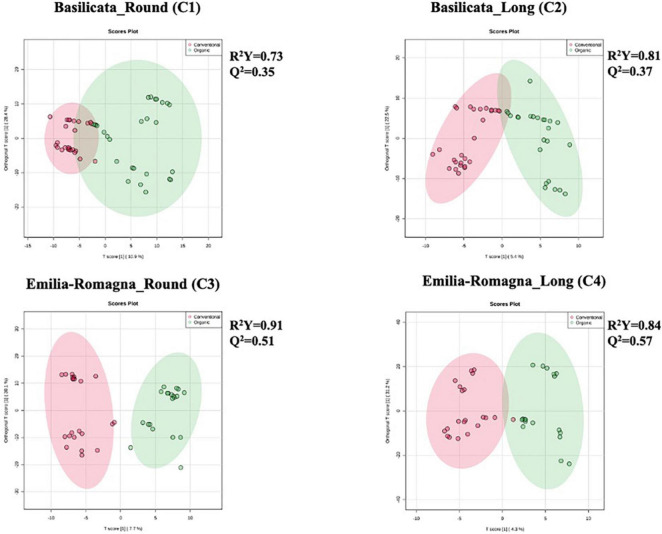
Different orthogonal projections to latent structures discriminant analysis (OPLS-DA) models relative to untargeted phytochemical profile of tomato samples, built considering the following comparisons: C1 (Basilicata; Cultivar: Round; Organic vs. Conventional), C2 (Basilicata; Cultivar: Long; Organic vs. Conventional), C3 (Emilia-Romagna; Cultivar: Round; Organic vs. Conventional), and C4 (Emilia-Romagna, Cultivar: Long; Organic vs. Conventional).

Starting from the complex phytochemical profile detected, a multivariate statistical approach based on unsupervised hierarchical cluster analysis (HCA) was used to outline the hierarchical contribution of the different factors in driving the differences observed for total phenolics and other compounds. Looking at the results, the FC-based heat map (Figure 1) showed that the tomato samples were grouped in two main clusters; the first one included five tomato samples from the Emilia Romagna region, except for three samples which clustered separately, and the second consisted in two sub-clusters. Interestingly, four tomato samples from Basilicata (growing year: 2013) demonstrated a similar phytochemical profile, independently from both the cultivar type and the farming system considered, thus suggesting a major effect of the geographical origin and growing year on the phytochemical profile, and thus supporting the results obtained from the *in vitro* spectrophotometric assays (Table 2). Regarding the other sub-cluster emerging from the heat map, neither the cultivar nor the farming system showed any distinctive profile able to justify a specific grouping. Starting from the unsupervised distribution observed in Figure 1, four different supervised OPLS-DA multivariate models were used to specifically dissect the weight of the farming system within each geographical origin and cultivar, independently from the growing season (Figure 2). The Variables Importance in Projection (VIP) approach was then used to select the most discriminating compounds of each comparison, considering a VIP score threshold >1.0. As shown in Figure 2, the OPLS-DA model built on tomato samples from Basilicata (models C1 and C2) showed limited prediction ability, characterized by Q^2^(cum) values lower than 0.5, although a separation trend could be highlighted along the latent vectors. Completely different results were outlined when considering the Emilia Romagna region (models C3 and C4), where the farming system was found to affect the phytochemical profile of the two cultivars under investigation, being the prediction ability values higher than 0.5 (namely 0.51 for the cultivar Round and 0.57 for the cultivar Long). Overall, the most discriminants compounds for each comparison were polyphenols (phenolic acids and flavonoids), vitamins, and carotenoids, recording a VIP score >1.9 (Supplementary Table 1). The four best discriminant compounds of the OPLS-DA model C1 were citramalate (VIP score = 2.116), hydroquinidine (VIP score = 2.046), L-ascorbic acid (VIP score = 1.998), and arbutin (VIP score = 1.975). For model C2 the best discriminants were *p*-coumaroyl tyrosine (VIP score = 2.526), 6″-*O*-malonyldaidzin (VIP score = 2.368), pterostilbene (VIP score = 2.336), and epirosmanol (VIP score = 2.239), whereas mevalonic acid (VIP score = 2.482), quercetin (VIP score = 2.394), rosmanol (VIP score = 2.352), and homovanillic acid (VIP score = 2.270) were identified for the OPLS-DA model C3. Finally, (9*Z*,9’*Z*)-7,7’,8,8’-tetrahydrolycopene (VIP score = 2.741), ligstroside (VIP score = 2.694), protocatechuic acid 4-*O*-glucoside (VIP score = 2.651), and 4,5-dicaffeoylquinic acid (VIP score = 2.627) were selected for the OPLS-DA model C4.

Two Venn diagram analyses were then carried out to understand the exclusive and common discriminant markers of the farming system, for Basilicata and Emilia-Romagna regions respectively (Supplementary Figures 2A,B, respectively). Regarding the first comparison (Supplementary Figure 2A) built considering tomato samples from the Basilicata region, we found 83 metabolites (24.7%) shared by the two cultivars, with 135 and 118 metabolites exclusively characterizing the Round and Long cultivars, respectively. A similar result was outlined by the Venn diagram on the second comparison (Supplementary Figure 2B, Emilia-Romagna region), with only 26.7% of VIP markers of the farming system being shared between the two cultivars. Overall, the multivariate statistical approaches revealed that the phytochemical profile of our tomato fruits was hierarchically more affected by the origin and the cultivar, than by the farming system. In fact, the “terroir effect,” determined by genotype *x* environment interaction, is known to play a central role in shaping the phytochemical profile of vegetables (Lucini et al., 2020; Suliman et al., 2021).

### Impact of the Farming Systems (Organic vs. Conventional) on the Phytochemical Profile of Tomato Fruits

**FIGURE 3:**
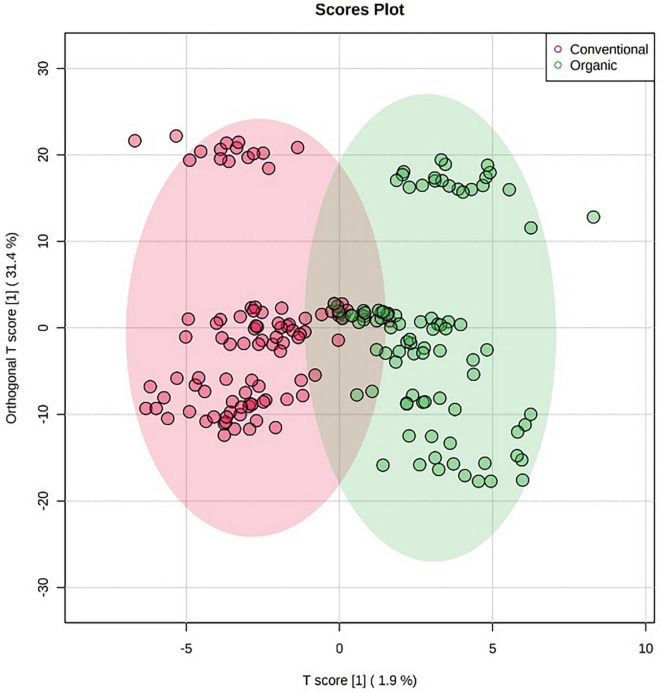
Orthogonal projections to latent structures discriminant analysis (OPLS-DA) score plot supervised modeling relative to untargeted metabolomics profile of tomato samples, built specifically considering the impact of farming system (i.e., organic *vs.* conventional) on the phytochemical profiles of tomato samples.

**FIGURE 4:**
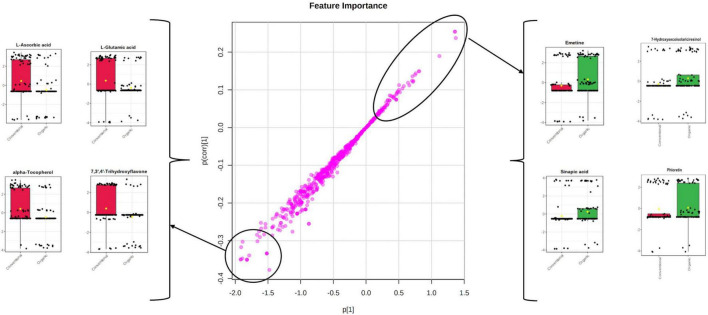
S-plot following the orthogonal projections to latent structures discriminant analysis (OPLS-DA) model on the different farming systems. The most discriminant features are highlighted, together with their Log2 fold-change variations.

Orthogonal projections to latent structures discriminant analysis multivariate modeling is a supervised approach having the ability to separate the predictive variability, i.e., the variability specifically related to the factor(s) to be modeled, from non-predictive (orthogonal) variability. Given this, the last OPLS-DA model was built to exclusively highlight the impact of farming systems on the different tomato cultivars, irrespectively from the origin, cultivar, and growing years. The corresponding OPLS-DA score plot is reported as Figure 3, while its corresponding S-plot showing the discriminant features is provided as Figure 4. As expected, the score plot on the farming system showed a high degree of variability, although a separation trend could be highlighted. The prediction model was characterized by the following parameters: 0.74 for the goodness-of-fitting (R^2^Y) and 0.41 for the goodness-of-prediction (Q^2^cum), thus outlining a good correlation but a moderate prediction ability. This pattern is coherent with previous results, where different and hierarchically more relevant “confounding factors” were shown to shape the metabolomic profile of tomato berries. Nonetheless, the corresponding S-plot following OPLS-DA was produced to highlight the potential candidate biomarkers for discriminating organic *vs.* conventional tomato samples. In particular, the coordinates in the lower-left quadrant were metabolites significantly increased in the conventional group, compared to the organic farming group, while those in the upper-right quadrant represent decreased metabolites.

Interestingly, the S-plot revealed that some of the most important variables able to characterize the tomato samples belonging to the conventional farming were L-ascorbic acid, L-glutamic acid, alpha-tocopherol, and 7,3′,4′-trihydroxyflavone (Figure 4), whilst among the markers better characterizing the organic farming we found emetine, 7-hydroxysecoisolariciresinol, sinapic acid, and phloretin (Figure 4). Environmental factors represent a powerful lever to modulate the concentrations in phytochemicals. Biotic or abiotic factors can promote oxidative stress in plants, triggering oxidative signaling and leading to the accumulation of secondary metabolites. Synthetic pesticides are perceived as xenobiotics by plants, representing a metabolic cost and triggering oxidative stress (Ganugi et al., 2021). Despite some effects provided by the pesticides used in organic farming (including copper) cannot be ruled out, the lack of synthetic pesticides in organic farming leads plants to accumulate secondary metabolites known as phytoalexins, to protect themselves against phytopathogens (Martínez Bueno et al., 2018). Several phenolics have been reported to act as plant phytoalexins and may be modulated by the farming system. The hypothesis that oxidative stress is involved in the modulation of phytochemicals in fruits and vegetables from organic farming was previously postulated (Oliveira et al., 2013). These authors showed that that tomato fruits from organic farming experienced stressing conditions that resulted in oxidative stress and the accumulation of higher concentrations of some phytochemicals contributing to nutritional quality (including phenolic compounds).

Our experimental conditions found that tomato samples belonging to conventional farming systems were characterized by higher levels of L-ascorbic acid and alpha-tocopherol, two key compounds involved in the antioxidant regulatory mechanisms. Regarding the accumulation of L-ascorbic acid, several authors have previously observed a controversial trend. In this regard, some authors (Vinha et al., 2014), found considerably higher levels of ascorbic acid under organic (23–30% increased) compared to conventional farming. Other authors, such as Hallmann (2012), have also observed this effect, but much more limited, whereas Juroszek et al. (2009) found no significant differences. On the other hand, Rossi et al. (2008) observed lower levels of vitamin C contents in tomato grown under organic farming systems, compared to conventional farming. A reduction in the ascorbate pool is expected when oxidative damage occurs; however, as reported by Dumas et al. (2003) other factors such as the nitrogen-based fertilization can reduce ascorbic acid synthesis, probably because lower levels of foliage growth require the shading of fruits, which triggers less ascorbic acid synthesis. In the study by Rossi et al. (2008), the organic tomatoes received more N-based fertilizers, thus determining an inverse and significant correlation between protein content and vitamin C levels, corresponding to an inverse relationship between N availability and vitamin C synthesis. Accordingly, in a previous work on our tomato samples, δ^15^N isotope was a main discriminant between conventional and organic agricultural practices, even if this parameter alone was not able to completely distinguish the farming systems (Bontempo et al., 2020). Therefore, taken together, our findings confirm the information available in the literature that L-ascorbic acid content is the result of different factors interacting in a complex manner. The farming system alone cannot fully justify the actual levels of L-ascorbic acid.

The presence of L-glutamic acid as a discriminant marker of the conventional farming system could be directly correlated to the choice of the fertilizer and other agricultural management practices that are critical in growing tomatoes and will differ depending on whether the tomatoes are grown organically or conventionally. The roots of non-N_2_-fixing plants take up nitrogen from the soil as either ammonium (NH_4_^+^) or nitrate (NO_3_^–^), and glutamate has a pivotal role in the downstream glutamine synthetase/glutamate synthase (GS-GOGAT) organication process. NH_4_^+^, once uptaken by the roots, is metabolized by glutamine synthetase and enters directly in the amino acids’ metabolism of the plant, whilst NO_3_^–^ is sequentially reduced by nitrate and nitrite reductases to nitrite and then ammonia. Through the assimilation of ammonia, glutamine synthetase produces the first amino acid, glutamine, and plays a central role in the amino acids’ assimilation. Thereafter, plants produce glutamate and then other amino acids through transamination reactions. It can be postulated that the differences in glutamate might represent a consequence of the different fertilization processes allowed in organic *vs.* conventional farming systems.

Regarding the last two discriminant compounds of the conventional farming systems selected by using the S-plot, the antioxidants alpha-tocopherol (a biologically active vitamer of vitamin E) and polyphenols were again pointed out. The factors that influence the concentration of phytonutrients such as carotenoids, tocopherols, and vitamin C in tomato include the maturity level of the fruit at the time of harvest, together with the agronomic, geographical, and environmental conditions as previously discussed. Among the variables possessing the highest discriminant power, we observed the phenolic compound 7,3′,4′-trihydroxyflavone. Also known as 5-deoxykaempferol, it belongs to flavonols, and was previously studied a potential therapeutic role by targeting multiple signaling pathways in skin cancer (Lee et al., 2010). No comprehensive information regarding this phenolic compound is available in the literature regarding its ability to discriminate among different farming systems. It is also important to highlight that, before harvest, the temperature is reported to profoundly affect the content of the different forms of α-tocopherol (free and esterified), similarly to what is generally observed on carotenoids. Such similarity is expected since carotenoids and tocopherols are lipid-soluble compounds synthesized by the same pathway in the membranes of the plant chromoplastids (Rath et al., 2020). Tocopherols contribute to seed longevity, seedling development, and protection of the photosynthetic apparatus against oxidative stress (Mène-Saffrané and DellaPenna, 2010). The involvement of polar and non-polar redox-related compounds, all being down-accumulated in tomato grown under organic farming (Figure 4), indicates that this latter condition may be related to oxidative imbalance in plant. This point is consistent with the hypersensitive response that plants activate in response to pathogens, a process triggered by the NO signaling cascade that involves peroxynitrite and reactive oxygen intermediates. The corresponding oxidative burst pivotally activates defense in the distal portions of the plant and ultimately results in systemic acquired resistance (Noman et al., 2020). It can be postulated that organically grown tomato may have experienced a higher pest pressure, or an improved diversity of the microbial community (Gu et al., 2019), that elicited systemic acquired resistance at the expense of antioxidant compounds.

Finally, we found several secondary metabolites among the marker compounds for the organic farming system. In this regard, emetine (an isoquinoline alkaloid) was highly discriminant for the organic group. This compound is described to possess antiviral and antiparasitic activity, as well as to regulate several genes (Akinboye and Bakare, 2011). Previous works pointed out that the biosynthesis of alkaloids is triggered according to the farming systems adopted (Martínez Bueno et al., 2018). Koh et al. (2013), reported that tomatine content in the same variety of tomatoes harvested from plants grown under “organic” conditions was about twice that of plants grown under conventional conditions. In our experimental conditions, other alkaloid compounds, such as koumidine and conessine, were pointed out together with emetine (not reported). Interestingly, alkaloids have been linked to hypersensitive response in plants, where they act as phytoalexins providing antimicrobial activity (Zehra et al., 2021). Nonetheless, a large part of the discriminant compounds highlighted by the S-plot following OPLS-DA modeling belonged to phenolics. Among others, a phenolic acid (sinapic acid), a lignan (7-Hydroxysecoisolariciresinol), and a flavonoid (phloretin) showed the largest accumulations. Phloretin is a known dihydrochalcone flavonoid of tomato, ranging in our experiments from 186 to 1,677 μg/kg FW in organic tomato samples to 20–73 μg/kg FW in conventional tomatoes. The accumulation of phloretin in organic tomato has also been reported by Anton et al. (2014). The accumulation of sinapic acid is also consistent with systemic acquired resistance (Wang et al., 2020), since this compound is included among syringyl lignin monomers triggered by the hypersensitive response in plants. Finally, also the lignans accumulation we observed in organically grown tomatoes can be related to plant response to stress (Xiao et al., 2020) and secoisolariciresonl in particular to biotic stress (Sánchez-Elordi et al., 2020).

## Conclusion

The controversial information in scientific literature about the health-promoting effects of organic foods, beyond the more consolidated food safety aspects like the absence of residues from chemical pesticides, may be explained by the different factors that affect plant functional traits at multiple levels. In our work, an experimental design accounting for cultivar-, seasonality-, and terroir-specific effects has been considered in addition to organic *vs.* conventional farming. Tomato has been used as a model crop, because of its broad secondary metabolism and diverse phytochemical constituents (including lipophilic and polar compounds). Here we provide evidence that, under our conditions, the combined effect of genotype (cultivar) and environment (pedo-climatic conditions and seasonality) were hierarchically prevalent over the cultivation system. These factors were found to significantly shape the metabolomic profile of tomato fruits and, consequently, their antioxidant and enzyme inhibition capacities. Notwithstanding, when supervised multivariate statistics were used to dissect the different contributions to the overall variability, some effects of the farming system at metabolome level could be highlighted. Overall, the changes elicited by organic farming could be ascribed to the elicitation of systemic acquired resistance, to include ascorbate and tocopherols decrease possibly triggering the accumulation of phytoalexins and lignin-intermediates (alkaloids, phenylpropanoids, and lignans). Overall, our findings suggest that all the factors under study must be considered when the functional traits of plant-based foods are to be evaluated, with the farming system not being the prevalent factor. The intricate interaction of cultivar and environment, followed by agronomic factors, may shape the actual health-promoting effects of vegetables. The information we provided worth to be investigated further, by including different plant species and/or different edaphic conditions, to eventually strengthen our findings. At the same time, despite being outside the aim of this work, food safety aspects and sustainability issues are additional key aspects to be considered when organic and conventional farming are compared.

## Data Availability Statement

The original contributions presented in the study are included in the article/Supplementary Material, further inquiries can be directed to the corresponding author.

## Author Contributions

LL designed the study, in cooperation with LB and FC. GR, BS, GZ, and PB carried out the experimental parts, contributed to data interpretation, and drafted and revised the manuscript. LL and GR developed the mass spectrometric method, performed statistics, and drafted the manuscript. MT, LL, LB, and FC drafted and critically revised the manuscript. All authors contributed to revise work critically, gave the final approval of the version to be published, and agreed on all aspects of the work.

## Conflict of Interest

PB was employed by company OloBion–OMICS LIFE LAB. The remaining authors declare that the research was conducted in the absence of any commercial or financial relationships that could be construed as a potential conflict of interest.

## Publisher’s Note

All claims expressed in this article are solely those of the authors and do not necessarily represent those of their affiliated organizations, or those of the publisher, the editors and the reviewers. Any product that may be evaluated in this article, or claim that may be made by its manufacturer, is not guaranteed or endorsed by the publisher.

## Footnotes

1
http://phenol-explorer.eu/

2
www.foodb.ca

3
https://www.metaboanalyst.ca/

## References

[B1] Ahmed S. Khan S. T. Zargaham M. K. Khan A. U. Khan S. Hussain A. (2021). Potential therapeutic natural products against Alzheimer’s disease with reference of Acetylcholinesterase. *Biomed. Pharmacother.* 139:111609. 10.1016/j.biopha.2021.111609 33915501

[B2] Akinboye E. S. Bakare O. (2011). Biological activities of emetine. *Nat. Prod. J*. 4 8–15. 10.2174/1874848101104010008

[B3] Anton D. Matt D. Pedastsaar P. Bender I. Kazimierczak R. Roasto M. (2014). Three-year comparative study of polyphenol contents and antioxidant capacities in fruits of tomato (*Lycopersicon esculentum* mill.) cultivars grown under organic and conventional conditions. *J. Agric. Food Chem*. 62 5173–5180. 10.1021/jf500792k 24811708

[B4] Bontempo L. van Leeuwen K. A. Paolini M. Holst Laursen K. Micheloni C. Prenzler P. D. (2020). Bulk and compound-specific stable isotope ratio analysis for authenticity testing of organically grown tomatoes. *Food Chem*. 318:126426. 10.1016/j.foodchem.2020.126426 32135420

[B5] Caris-Veyrat C. Amiot M. J. Tyssandier V. Grasselly D. Buret M. Mikolajczak M. (2004). Influence of organic versus conventional agricultural practice on the antioxidant microconstituent content of tomatoes and derived purees; consequences on antioxidant plasma status in humans. *J. Agric. Food Chem.* 52, 6503–6509. 10.1021/jf0346861 15479014

[B6] Chaudhary P. Sharma A. Singh B. Nagpal A. K. (2018). Bioactivities of phytochemicals present in tomato. *J. Food Sci. Technol.* 55 2833–2849. 10.1007/s13197-018-3221-z 30065393 PMC6045986

[B7] Dumas Y. Dadomo M. Di Lucca G. Grolier P. (2003). Effects of environmental factors and agricultural techniques on antioxidant content of tomatoes. *J. Sci. Food Agric.* 83 369–382. 10.1002/jsfa.1370

[B8] Ganugi P. Miras-Moreno B. Garcia-Perez P. Lucini L. Trevisan M. (2021). Concealed metabolic reprogramming induced by different herbicides in tomato. *Plant Sci*. 303:110727. 10.1016/j.plantsci.2020.110727 33487335

[B9] Gu S. Hu Q. Cheng Y. Bai L. Liu Z. Xiao W. (2019). Application of organic fertilizer improves microbial community diversity and alters microbial network structure in tea (*Camellia sinensis*) plantation soils. *Soil Tillage Res*. 195:104356. 10.1016/j.still.2019.104356

[B10] Hallmann E. (2012). The influence of organic and conventional cultivation systems on the nutritional value and content of bioactive compounds in selected tomato types. *J. Sci. Food Agric*. 92 2840–2848. 10.1002/jsfa.5617 22351383

[B11] Hallmann E. Lipowski J. Marszałek K. Rembiałkowska E. (2013). The seasonal variation in bioactive compounds content in juice from organic and non-organic tomatoes. *Plant Foods Hum. Nutr.* 68 171–176. 10.1007/s11130-013-0352-2 23609833 PMC3659276

[B12] Jan R. Asaf S. Numan M. Lubna Kim K. M. (2021). Plant secondary metabolite biosynthesis and transcriptional regulation in response to biotic and abiotic stress conditions. *Agronomy* 11:968. 10.3390/agronomy11050968

[B13] Johansson E. Hussain A. Kuktaite R. Andersson S. C. Olsson M. E. (2014). Contribution of organically grown crops to human health. *Int. J. Environ. Res.Public Health* 11 3870–3893. 10.3390/ijerph110403870 24717360 PMC4025038

[B14] Juroszek P. Lumpkin H. M. Yang R. Y. Ledesma D. R. Ma C. H. (2009). Fruit quality and bioactive compounds with antioxidant activity of tomatoes grown on-farm: comparison of organic and conventional management systems. *J. Agric.Food Chem*. 57 1188–1194. 10.1021/jf801992s 19178281

[B15] Koh E. Kaffka S. Mitchell A. E. (2013). A long-term comparison of the influence of organic and conventional crop management practices on the content of the glycoalkaloid α-tomatine in tomatoes. *J. Sci. Food Agric*. 93 1537–1542. 10.1002/jsfa.5951 23138335

[B16] Lee K. M. Lee K. W. Byun S. Jung S. K. Seo S. K. Heo Y. S. (2010). 5-Deoxykaempferol plays a potential therapeutic role by targeting multiple signaling pathways in skin cancer. *Cancer Prev. Res*. 3 454–465. 10.1158/1940-6207.CAPR-09-0137 20233901

[B17] Lucini L. Rocchetti G. Trevisan M. (2020). Extending the concept of terroir from grapes to other agricultural commodities: an overview. *Curr. Opin. Food Sci*. 31 88–95. 10.1016/j.cofs.2020.03.007

[B18] Mabizela G. S. Muller M. de Beer D. van der Rijst M. Slabbert M. M. Joubert E. (2020). Effect of genotype and harvest season on quality characteristics of *Cyclopia subternata*: phenolic content and sensory profile. *S. Afr. J. Bot.* 132 491–501. 10.1016/j.sajb.2020.06.010

[B19] Martínez Bueno M. J. Díaz-Galiano F. J. Rajski Ł Cutillas V. Fernández-Alba A. R. (2018). A non-targeted metabolomic approach to identify food markers to support discrimination between organic and conventional tomato crops. *J. Chromatogr. A* 1546 66–76. 10.1016/j.chroma.2018.03.002 29526497

[B20] Mène-Saffrané L. DellaPenna D. (2010). Biosynthesis, regulation and functions of tocochromanols in plants. *Plant Physiol. Biochem*. 48 301–309. 10.1016/j.plaphy.2009.11.004 20036132

[B21] Mitchell A. E. Hong Y. J. Koh E. Barrett D. M. Bryant D. E. Denison R. F. (2007). Ten-year comparison of the influence of organic and conventional crop management practices on the content of flavonoids in tomatoes. *J. Agric. Food Chem.* 55 6154–6159. 17590007 10.1021/jf070344+

[B22] Noman A. Aqeel M. Qari S. H. Al Surhanee A. A. Yasin G. Alamri S. (2020). Plant hypersensitive response vs pathogen ingression: death of few gives life to others. *Microb. Pathog*. 145:104224. 10.1016/j.micpath.2020.104224 32360524

[B23] Novotná H. Kmiecik O. Gałazka M. Krtková V. Hurajová A. Schulzová V. (2012). Metabolomic fingerprinting employing DART-TOFMS for authentication of tomatoes and peppers from organic and conventional farming. *Food Addit. Contam. Part A Chem. Anal. Control Expo. Risk Assess*. 29 1335–1346. 10.1080/19440049.2012.690348 22813205

[B24] Oliveira A. B. Moura C. F. H. Gomes-Filho E. Marco C. A. Urban L. Miranda M. R. A. (2013). The impact of organic farming on quality of tomatoes is associated to increased oxidative stress during fruit development. *PLoS One* 8:e56354. 10.1371/journal.pone.0056354 23437115 PMC3577808

[B25] Rath S. Egei M. Horwh K. Andryie B. Daood H. G. (2020). EFFECT of different ecological conditions on content of phytonutrients in industrial tomatoes. *Acta Aliment*. 49 225–234. 10.1556/066.2020.49.2.12

[B26] Riahi A. Hdider C. Sanaa M. Tarchoun N. Ben Kheder M. Guezal I. (2009). Effect of conventional and organic production systems on the yield and quality of field tomato cultivars grown in Tunisia. *J. Sci. Food Agric.* 89 2275–2282. 10.1002/jsfa.3720

[B27] Rocchetti G. Senizza B. Putnik P. Bursać Kovačević D. Barba F. J. Trevisan M. (2019). Untargeted screening of the bound / free phenolic composition in tomato cultivars for industrial transformation. *J. Sci.Food Agric*. 99 6173–6181. 10.1002/jsfa.9889 31250429

[B28] Rocchetti G. Zengin G. Cakmak Y. S. Mahomoodally M. F. Kaya M. F. Alsheikh S. M. (2020). A UHPLC-QTOF-MS screening provides new insights into the phytochemical composition and biological properties of six Consolida species from Turkey. *Ind. Crop. Prod.* 158 112966. 10.1016/j.indcrop.2020.112966

[B29] Rossi F. Godani F. Bertuzzi T. Trevisan M. Ferrari F. Gatti S. (2008). Health-promoting substances and heavy metal content in tomatoes grown with different farming techniques. *Eur. J.Nutr.* 47 266–272. 10.1007/s00394-008-0721-z 18604621

[B30] Sánchez-Elordi E. Sterling R. M. Santiago R. de Armas R. Vicente C. Legaz M. E. (2020). Increase in cytotoxic lignans production after smut infection in sugar cane plants. *J. Plant Physiol*. 244:153087. 10.1016/j.jplph.2019.153087 31816510

[B31] Schmeda-Hirschmann G. Antileo-Laurie J. Theoduloz C. Jiménez-Aspee F. Avila F. Burgos-Edwards A. (2021). Phenolic composition, antioxidant capacity and α-glucosidase inhibitory activity of raw and boiled Chilean *Araucaria araucana* kernels. *Food Chem.* 350 129241. 10.1016/j.foodchem.2021.129241 33601092

[B32] Sharma S. Katoch V. Kumar S. Chatterjee S. (2021). Functional relationship of vegetable colors and bioactive compounds: implications in human health. *J. Nutr. Biochem*. 92:108615. 10.1016/j.jnutbio.2021.108615 33705954

[B33] Suliman S. Alemu A. Abdelmula A. A. Badawi G. H. Al-Abdallat A. Tadesse W. (2021). Genome-wide association analysis uncovers stable QTLs for yield and quality traits of spring bread wheat (*Triticum aestiv*um) across contrasting environments. *Plant Gene* 25:100269. 10.1016/j.plgene.2020.100269

[B34] Szabo K. Cătoi A. F. Vodnar D. C. (2018). Bioactive compounds extracted from tomato processing by-products as a source of valuable nutrients. *Plant Foods Hum. Nutr*. 73 268–277. 30264237 10.1007/s11130-018-0691-0

[B35] Tsugawa H. Cajka T. Kind T. Ma Y. Higgins B. Ikeda K. (2015). MS-DIAL: data-independent MS/MS deconvolution for comprehensive metabolome analysis. *Nat. Methods* 12 523–526. 10.1038/nmeth.3393 25938372 PMC4449330

[B36] Uysal S. Zengin G. Aktumsek A. Karatas S. (2016). Chemical and biological approaches on nine fruit tree leaves collected from the Mediterranean region of Turkey. *J. Funct. Foods* 22 518–532. 10.1016/j.jff.2016.02.006

[B37] Vinha A. F. Barreira S. V. P. Costa A. S. G. Alves R. C. Oliveira M. B. P. P. (2014). Organic versus conventional tomatoes: influence on physicochemical parameters, bioactive compounds and sensorial attributes. *Food Chem. Toxico*l. 67 139–144. 10.1016/j.fct.2014.02.018 24569070

[B38] Wang Y. Yang Q. Jiang H. Wang B. Bi Y. Li Y. (2020). Reactive oxygen species-mediated the accumulation of suberin polyphenolics and lignin at wound sites on muskmelons elicited by benzo (1, 2, 3)-thiadiazole-7-carbothioic acid S-methyl ester. *Postharvest Biol. Technol*. 170:111325. 10.1016/j.postharvbio.2020.111325

[B39] Xiao Y. Feng J. Li Q. Zhou Y. Bu Q. Zhou J. (2020). IiWRKY34 positively regulates yield, lignan biosynthesis and stress tolerance in Isatis indigotica. *Acta Pharm. Sin. B*. 10 2417–2432. 10.1016/j.apsb.2019.12.020 33354511 PMC7745056

[B40] Yu X. Meenu M. Xu B. Yu H. (2021). Impact of processing technologies on isoflavones, phenolic acids, and antioxidant capacities of soymilk prepared from 15 soybean varieties. *Food Chem.* 345:128612. 10.1016/j.foodchem.2020.128612 33352407

[B41] Zehra A. Raytekar N. A. Meena M. Swapnil P. (2021). Efficiency of microbial bio-agents as elicitors in plant defense mechanism under biotic stress: a review. *Curr. Res. Microb. Sci*. 2:100054. 10.1016/j.crmicr.2021.100054 34841345 PMC8610294

