# Supplementary material for: The Hierarchical Contribution of Organic vs. Conventional Farming, Cultivar, and Terroir on Untargeted Metabolomics Phytochemical Profile and Functional Traits of Tomato Fruits
Source: Front Plant Sci. 2022 Mar 25;13:856513. doi: 10.3389/fpls.2022.856513 (PMC8992384; doi:10.3389/fpls.2022.856513)
Supplement: Supplementary file 3 [file Data_Sheet_3.PDF]

Monthly weather conditions in the field locations of the study (average from the last 10 years)

Emilia Romagna

	January	February	March	April	May	June	July	August	september	October	November	December
Mean Temperature (°C)	3	4.7	9.2	13.3	17.9	22.6	25	24.5	19.6	14.5	8.7	3.7
minimum Temperature (°C)	-0.2	0.6	4.2	8.2	12.6	17.1	19.6	19.5	15.2	11	5.7	0.8
Max Temperature (°C)	7	9.3	14.3	18.2	22.8	27.6	30.1	29.5	24.2	18.5	12.1	7.3
Rain (mm)	62	65	71	96	96	75	55	73	95	109	117	71
Humidity (%)	83%	77%	71%	70%	67%	63%	58%	61%	67%	77%	84%	85%
rainy days (dd)	6	6	6	8	8	7	6	7	7	8	8	7
Hours of sun (h)	4.6	5.8	7.6	9	11	12.3	12.5	11	9	5.5	4	4.2

Basilicata

	January	February	March	April	May	June	July	August	september	October	November	December
Mean Temperature (°C)	5.4	5.9	9	12.5	17.1	22.1	24.9	24.9	19.7	15.5	10.9	6.6
minimum Temperature (°C)	2	1.9	4.5	7.4	11.5	15.8	18.5	18.8	14.9	11.2	7.2	3.1
Max Temperature (°C)	9.4	10.1	13.8	17.7	22.5	27.8	30.8	31	24.8	20.5	15.2	10.5
Rain (mm)	63	58	68	67	45	35	27	22	48	57	64	70
Humidity (%)	81%	78%	73%	69%	63%	53%	48%	49%	64%	73%	78%	82%
rainy days (dd)	8	8	8	8	6	4	3	4	6	6	7	8
Hours of sun (h)	5.4	5.9	7.6	9.3	11	12.3	12.4	11.6	9.3	7.4	6.3	5.4

source: *https://it.climate-data.org/* *Accessed: 22 Feb 2022*

Weather conditions (yearly basys and during the growing period for tomato) in the field locations of the study (average from the last 10 years)

Yearly			growing season (april-august)		
min	max	average	min	max	average
3	25	14	13.3	25	21
-0.2	19.6	10	8.2	19.6	15
7	30.1	18	18.2	30.1	26
55	117	82	55	96	79
58%	85%	72%	58%	70%	64%
6	8	7	6	8	7
4	12.5	8	9	12.5	11.2

Yearly			growing season (april-august)		
min	max	average	min	max	average
5.4	24.9	15	12.5	24.9	20
1.9	18.8	10	7.4	18.8	14
9.4	31	20	17.7	31	26
22	70	52	22	67	39
48%	82%	68%	48%	69%	56%
3	8	6	3	8	5
5.4	12.4	9	9.3	12.4	11.3
