# Supplementary material for: The Hierarchical Contribution of Organic vs. Conventional Farming, Cultivar, and Terroir on Untargeted Metabolomics Phytochemical Profile and Functional Traits of Tomato Fruits
Source: Front Plant Sci. 2022 Mar 25;13:856513. doi: 10.3389/fpls.2022.856513 (PMC8992384; doi:10.3389/fpls.2022.856513)
Supplement: Supplementary file 4 [file Data_Sheet_4.docx]

**Table S1.** Conditions for growing tomatoes in Emilia Romagna in years 2012 and 2013.

|  |  |  |  |  |  |
| --- | --- | --- | --- | --- | --- |
| Parameter |  | Emilia Romagna 2012 | | | |
|  |  |  |  |  |  |
| Tomato variety |  | organic long | organic round | conventional long | conventional round |
| Seed |  | organic | organic | conventional and treated | conventional and treated |
| Planting date |  | beginning May | beginning May | beginning May | beginning May |
| Soil type |  | clay | clay | clay | clay |
| Fertilizers |  | organic fertilizer (50kg N and 100kg P_2_O_5_) | farm-yard manure | mineral - organic fertilizer (100kg N and 150kg P_2_O_5_) | mineral - organic fertilizer (100kg N and 150kg P_2_O_5_) |
| Plant protection products |  | copper | copper | Propamocarb | Propamocarb and Imidaclorprid |
| Weed management |  | mechanical | mechanical | Pendimethalin and Metribuzin | Pendimethalin and Metribuzin |
| Preceding crop |  | luzerne | vegetables | luzerne | wheat |
| Harvesting date |  | mid September | mid September | mid September | mid September |
| Harvesting quantity |  | 60t/ha | 50t/ha | 70t/ha | 70t/ha |
|  |  |  |  |  |  |

|  |  |  |  |  |  |
| --- | --- | --- | --- | --- | --- |
| Parameter |  | Emilia Romagna 2013 | | | |
|  |  |  |  |  |  |
| Tomato variety |  | organic long | organic round | conventional long | conventional round |
| Seed |  | organic | organic | conventional and treated | conventional and treated |
| Planting date |  | beginning May | end April | beginning May | beginning May |
| Soil type |  | clay | clay | clay | clay |
| Fertilizers |  | farm-yard manure | farm-yard manure | mineral - organic fertilizer (100kg N and 150kg P_2_O_5_) | mineral - organic fertilizer (100kg N and 150kg P_2_O_5_) |
| Plant protection products |  | copper | copper | Propamocarb and Imidaclorprid | Propamocarb and Imidaclorprid |
| Weed management |  | mechanical | mechanical | Pendimethalin and Metribuzin | Pendimethalin and Metribuzin |
| Preceding crop |  | vegtables | luzerne | wheat | wheat |
| Harvesting date |  | end August | end August | end August | end August |
| Harvesting quantity |  | 60t/ha | 50t/ha | 90t/ha | 90t/ha |
|  |  |  |  |  |  |

**Table S2.** Conditions for growing tomatoes in Basilicata in years 2012 and 2013.

|  |  |  |  |  |
| --- | --- | --- | --- | --- |
| Parameter | Basilicata 2012 | | | |
|  |  |  |  |  |
| Tomato variety | organic long | organic round | conventional long | conventional round |
| Seed | organic | organic | conventional and treated | conventional and treated |
| Planting date | mid May | mid May | mid May | mid May |
| Soil type | loamy, medium soil matter | loamy, medium soil matter | clay-loamy, high pH | clay-loamy, high pH |
| Fertilizers | organic fertilizer (24kg N and 60kg P_2_O_5_) | organic fertilizer (24kg N and 60kg P_2_O_5_) | mineral-organic fertilizer (6kg N and 44kg P_2_O_5_ + foliar fertilization with 6kg N + auxines) | mineral-organic fertilizer (6kg N and 44kg P_2_O_5_ + foliar fertilization with 6kg N + auxines) |
| Plant protection products | garlic extracts, copper, sulphur, propolis and bentonitis | garlic extracts, copper, sulphur, propolis and bentonitis | Propamocarb and Imidaclorprid | Propamocarb and Imidaclorprid |
| Weed management | mechanical | mechanical | Pendimethalin and Metribuzin | Pendimethalin and Metribuzin |
| Preceding crop | cover crop (hairy vetch and oat) | cover crop (hairy vetch and oat) | faba bean | faba bean |
| Harvesting date | end August | end August | mid August | mid August |
| Harvesting quantity | 30t/ha (average) | 28t/ha (average) | 80t/ha | 70t/ha |
|  |  |  |  |  |

|  |  |  |  |  |  |
| --- | --- | --- | --- | --- | --- |
| Parameter |  | Basilicata 2013 | | | |
|  |  |  |  |  |  |
| Tomato variety |  | organic long | organic round | conventional long | conventional round |
| Seed |  | organic | organic | conventional and treated | conventional and treated |
| Planting date |  | mid May | mid May | end April | end April |
| Soil type |  | loamy, medium soil matter | loamy, medium soil matter | clay-loamy, high pH | clay-loamy, high pH |
| Fertilizers |  | organic fertilizer (20kg N and 20kg P_2_O_5_ 32kg K_2_O) | organic fertilizer (20kg N and 20kg P_2_O_5_ 32kg K_2_O) | Farmyard manure + 20kg CaNO_2_, 20kg P_2_O_5,_ 20kg KNO_3_ | Farmyard manure + 20kg CaNO_2_, 20kg P_2_O_5,_ 20kg KNO_3_ |
| Plant protection products |  | garlic extracts,copper, sulphur, propolis, neem oil | garlic extracts, copper, sulphur, propolis, neem oil | Imidaclorprid, Cimoxanil, Metalaxil, Copper, Clorpirifos, Tebuconazolo, Idoxarb and Emamectina Benxzoate | Propamocarb and Imidaclorprid |
| Weed management |  | mechanical | mechanical | Oxadiazon, Rimsulfuron | Oxadiazon, Rimsulfuron |
| Preceding crop |  | durum wheat | durum wheat | corn | corn |
| Harvesting date |  | end August | end August | mid August | mid August |
| Harvesting quantity |  | 30t/ha (average) | 20t/ha (average) | 50t/ha | 50t/ha |
|  |  |  |  |  |  |

**Table S3.** Average precipitation (mm) and temperature (°C) in growing localities.

|  |  |  |  |  |  |  |  |  |  |  |
| --- | --- | --- | --- | --- | --- | --- | --- | --- | --- | --- |
|  |  | Average rain (mm) | | | |  | Average daily temperatures (°C) | | | |
|  |  |  |  |  |  |  |  |  |  |  |
|  |  | Emilia Romagna 2012 | Emilia Romagna 2013 | Basilicata 2012 | Basilicata 2013 |  | Emilia Romagna 2012 | Emilia Romagna 2013 | Basilicata 2012 | Basilicata 2013 |
|  |  |  |  |  |  |  |  |  |  |  |
|  |  |  |  |  |  |  |  |  |  |  |
| May |  | 93 | 120 | 20 | 10 |  | 20.6 | 18.5 | 17.7 | 19.2 |
| June |  | 8 | 23 | 7 | 55 |  | 27.9 | 24.3 | 25.8 | 23.2 |
| July |  | 1 | 12 | 23 | 97 |  | 29.7 | 28.6 | 28.3 | 26.0 |
| August |  | 2 | 26 | 2 | 30 |  | 30.0 | 26.9 | 27.7 | 27.2 |
| total |  | 104 | 181 | 52 | 192 |  |  |  |  |  |
|  |  |  |  |  |  |  |  |  |  |  |

Sources meteo data:

for Basilicata (BAS) Servizio Agrometeorologico Lucano-ALSIA

for Emilia Romagna (ER) ARPA Emilia Romagna
