# Supplementary material for: The Hierarchical Contribution of Organic vs. Conventional Farming, Cultivar, and Terroir on Untargeted Metabolomics Phytochemical Profile and Functional Traits of Tomato Fruits
Source: Front Plant Sci. 2022 Mar 25;13:856513. doi: 10.3389/fpls.2022.856513 (PMC8992384; doi:10.3389/fpls.2022.856513)

**Supplementary figure 1.** Pie chart showing the major classes annotated in tomato samples according to the untargeted UHPLC-QTOF-MS approach.


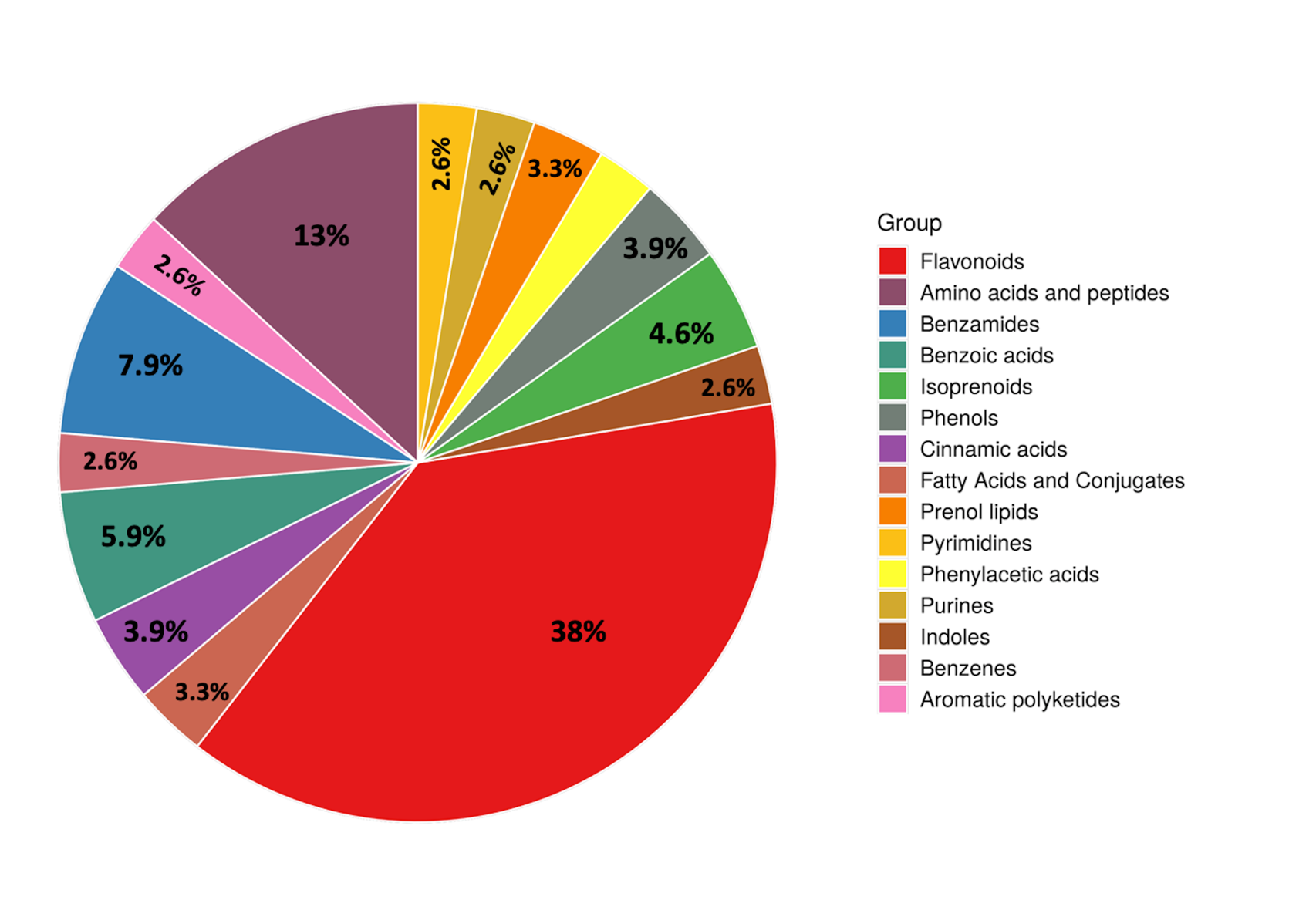


**Supplementary figure 2.** Venn diagram considering the VIP marker compounds of the different OPLS-DA model built to highlight interaction between terroir conditions and farming systems: C1 (Basilicata; Cultivar: Round; Organic vs Conventional), C2 (Basilicata; Cultivar: Long; Organic vs Conventional), C3 (Emilia-Romagna; Cultivar: Round; Organic vs Conventional), and C4 (Emilia-Romagna, Cultivar: Long; Organic vs Conventional).


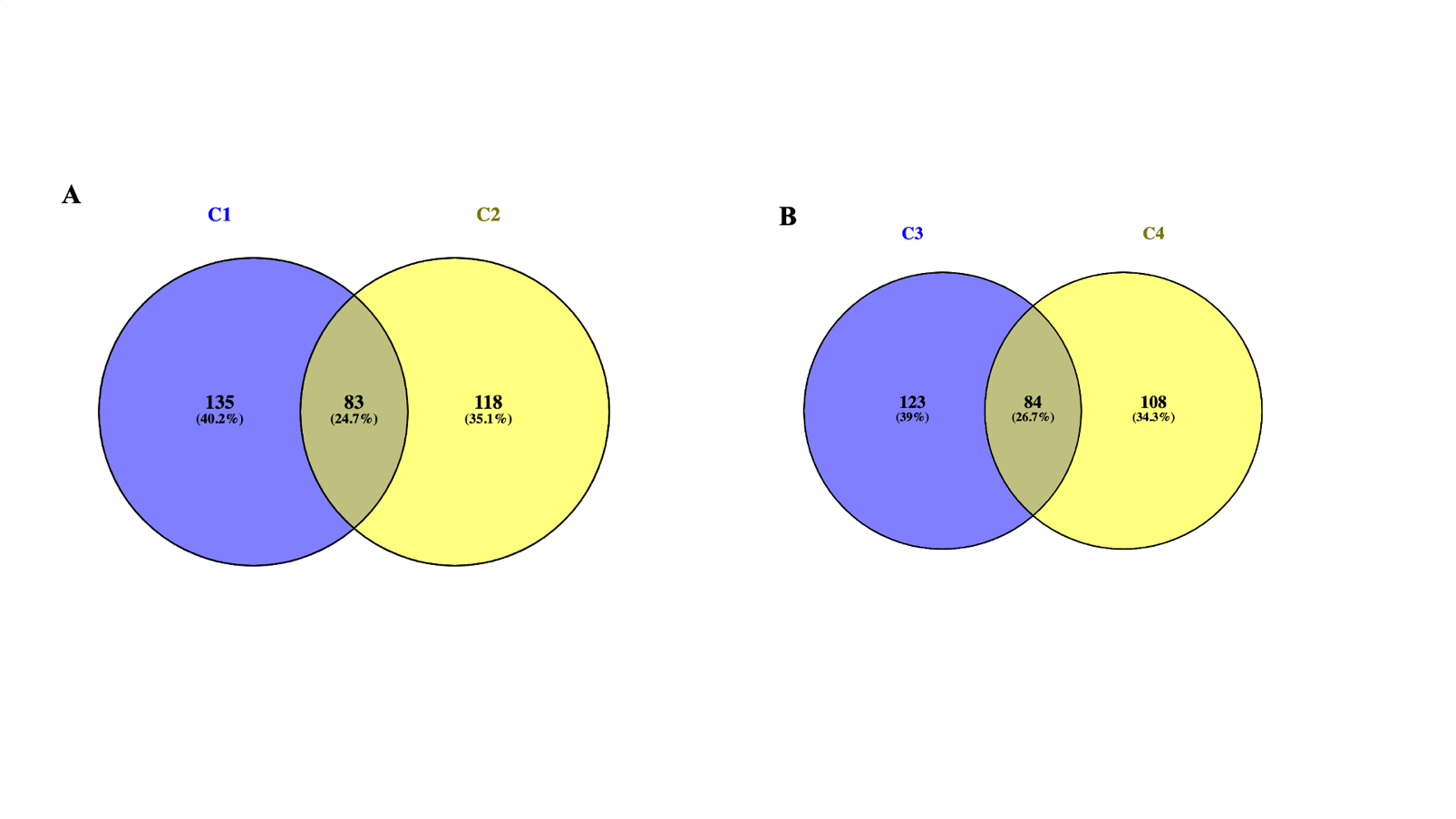

Supplement: Supplementary file 5 [file Data_Sheet_5.docx]
